# Supplementary material for: Metformin and the Development of Asthma in Patients with Type 2 Diabetes
Source: Int J Environ Res Public Health. 2022 Jul 5;19(13):8211. doi: 10.3390/ijerph19138211 (PMC9266193; doi:10.3390/ijerph19138211)
Supplement: Supplementary file 1 [file ijerph-19-08211-s001.zip › ijerph-1773008-supplementary.pdf]

# Supplementary Material

**Table S1**. Diseases and related ICD-9-CM, ICD-10-CM codes.

| Disease | ICD-9-CM Codes | ICD-10-CM Codes |
|---|---|---|
| Type 2 diabetes | 250.xx, except 250.1x | E11 |
| Type 1 diabetes | 250.1x | E10 |
| Asthma | 493 | J45 |
| Dialysis | V56.0, V56.8, V45.1 | Z49.31, Z49.32, Z99.2 |
| Hepatic failure | 570, 572.2, 572.4, 572.8 | K72.00, K72.01, K72.10, K72.11, K72.90, K76.2, K72.90, K72.91, K76.7, K76.81 |
| Overweight | 278.02, 783.1, V85.2 | R63.5 |
| Obesity | 278.00, 649.1, V77.8, V85.3 | E66.09, E66.1, E66.8, E66.9, Z13.89 |
| Severe obesity | 278.01, 649.2, V45.86, V85.4 | E66.01, E66.2 |
| Smoking status | 305.1, 649.0, V15.82 | F17.200, F17.201, F17.210, F17.220, F17.221, F17.290, F17.291, Z87.891 |
| Alcohol-related disorders | 291, 303, 305.0, 571.0-571.3, V11.3, V79.1 | F10, K70.40, K70.41, K70.9 |
| Hypertension | 401–405, A26 | I10, I11, I12, I13, I15, N26 |
| Dyslipidemia | 272 | E71.30, E71.31, E71.32, E71.39, E75.21, E75.22, E75.23, E75.24, E75.25, E75.29, E75.3, E75.4, E75.5, E75.6, E77, E78.0, E78.1, E78.2, E78.3, E78.4, E78.5, E78.6, E78.70, E78.71, E78.72, E78.79, E78.8, E78.9 |
| Coronary artery disease | 410-414 | I20, I21, I22, I24, I25.1, I25.2, I25.3, I25.4, I25.5, I25.6, I25.7, I25.81, I25.82, I25.83, I25.84, I25.89, I25.9 |
| Chronic kidney disease | 250.4x, 403.xx, 404.xx, 585.xx, 586.xx, 581.8x, 791.0x, 593.9x, V42.0x, V45.1x, V56.0x, V56.8x, 39.27, 39.42, 39.43, 39.49, 39.50, 39.53, 39.93, 39.94, or 39.95 | E10.2, E10.65, E11.2, E11.65, E13.2, I12, I13, N03, N08, E10.21, E11.21, N05, N06, N07, N14, N15.0, N15.8, N15.9, N16, N17.1, N17.2, N18, N19, Z94.0, Z49.31, Z49.32, Z99.2, Z94.0 |
| Stroke | 430-438 | G45.0, G45.1, G45.2, G45.3, G45.4, G45.8, G45.9, G46, I60, I61, I62, I63, I65, I66, I67.0, I67.1, I67.2, I67.3, I67.4, I67.5, I67.6, I67.7, I67.8, I67.9, I68, I69 |
| Atrial fibrillation | 427 | I45.0, I45.1, I45.2, I45.3, I45.4, I45.5, I45.6 |
| Heart failure | 398.91, 402.01, 402.11, 402.91, 404.01, 404.03, 404.11, 404.13, 404.91, 404.93, 428, 429.4 | I09.81, I11.0, I13.0, I13.2, I50, I97.0, I97.110, I97.111, I97.120, I97.121, I97.130, I97.131, I97.190, I97.191 |

| | | |
|---|---|---|
| Peripheral arterial occlusive disease | 440.0, 440.20, 440.21, 440.22, 440.23, 440.24, 440.3, 440.4, 443.9, 443.81, 443.89 | I70.2, I70.92, I75.0, I73.9 |
| Chronic obstructive pulmonary disease | 491, 492, or 496 | J41, J42, J44, J43, or J44.9 |
| Rheumatoid arthritis | 714.0 | M06.9 |
| Systemic lupus erythematosus | 710.0 | M32.10 |
| Liver cirrhosis | 571.5, 571.2, 571.6 | K70.2, K70.30, K70.31, K74.0, K74.1, K74.2, K74.60, K74.69, K74.3, K74.4, K74.5 |
| Cancers | 140-178, 190-199, 209 | C00-C63, C69-C80, C7A-C7B |
| Psychosis | 290–299, except 290,290.4, 291.2, 292.82 | F20-29 |
| Depression | 311 | F32, F33 |
| Dementia | 290,290.4, 291.2, 292.82 and 331 | F03.90, F05, F02.80, F02.81, F01.50, F01.51, G30 |
